# Preventing maternal deaths from hypertensive disorders of pregnancy through strengthening of primary care services

**DOI:** 10.4102/phcfm.v18i1.5364

**Published:** 2026-05-06

**Authors:** Olive P. Khaliq, Jagidesa Moodley, Nnabuike C. Ngene

**Affiliations:** 1 Department of Paediatrics and Child Health, Faculty of Health Sciences, University of the Free State, Bloemfontein, South Africa; 2 Women’s Health and HIV, Department of Obstetrics and Gynaecology, College of Health Sciences, University of KwaZulu-Natal, Durban, South Africa; 3 Department of Obstetrics and Gynaecology, Faculty of Health Sciences, University of the Witwatersrand, Johannesburg, South Africa

## Introduction

Hypertensive disorders of pregnancy (HDP), in particular severe preeclampsia and eclampsia, remain a leading preventable cause of maternal mortality and stillbirths in South Africa. The South African 2023 annual Saving Mothers Report and the triennial Saving Mothers Report covering the years 2020–2022 identify HDP as a major direct cause of maternal mortality.^1,2^ Interestingly, the 2023 annual Saving Mothers Report shows that the in-hospital maternal mortality ratio (iMMR) from HDP has declined.^1^ This is a commendable achievement; however, we argue that the decline can be improved because maternal deaths from HDP are largely preventable.

The iMMR from the top causes of maternal death in South Africa in the last 5 years are shown in Table 1. Table 1 also shows that the overall change in iMMR falls below the global projected expectation (of 7.5% reduction per annum) to achieve the Sustainable Development Goal of 70 per 100 000 live births by the year 2030.^3^ The overall iMMR must decrease by a further 32 per 100 000 over the next 7-years to achieve this target. Most women seeking antenatal care are first seen in public sector primary care and district hospitals, especially in rural areas of South Africa.^4,5^ Ironically, most of the deaths occurred in women who received early antenatal care.^6,7^ Nevertheless, further decreases in deaths from HDP as a key component of this will require strengthening of primary care services.

**TABLE 1:** In-hospital maternal mortality ratio in South Africa and its underlying causes from the year 2019 to 2023.

Causes	Yearly iMMR per 100 000 live births					Pattern of change
	2019	2020	2021	2022	2023	
Hypertensive disorders	19.4	17.9	18.5	17.2	16.9	Decreased
Non-pregnancy infection	20.0	31.1	55.2	18.6	17.4	Decreased
Obstetric haemorrhage	18.1	19.3	23.3	16.7	16.5	Decreased
Medical and surgical disorders	15.6	17.7	18.7	14.5	17.2	Increased
iMMR from all causes of maternal deaths	98.8	115.6	146.6	100.1	102.1	Increased

*Source*: South African Department of Health. National Committee for Confidential Enquiry into Maternal Deaths: Annual report for 2023 [homepage on the Internet]. 2024 [cited 2025 Dec 13]. Available from: https://www.health.gov.za/wp-content/uploads/2024/10/Saving-Mothers-Report-2023.pdf^1^

iMMR, in-hospital maternal mortality ratio.

## Avoidable factors

The preventable maternal deaths factors in primary care clinics include lack of administration of antihypertensive medication and magnesium sulphate prophylaxis when indicated, as well as noncompliance with other aspects of the national guidelines, namely, the failure to initiate aspirin prophylaxis in women at risk.^7^ Improving the quality of primary antenatal care can prevent later in-hospital mortality.^8^ Notably, this does not underrate the contributions of avoidable factors at higher levels of care. Furthermore, the national guidelines of 2019 on management of HDP also highlights gaps in primary care services, including delayed recognition of risk factors, failure to escalate clinical care to an experienced colleague in the same facility when indicated, poor resuscitation, and delayed referral to higher levels of healthcare.^7,9^ We envisage that addressing these gaps will further reduce maternal deaths because of HDP.

It appears that the 2019 national guidelines on HDP management,^9^ which provides evidence-based clinical recommendations for primary care clinics and district hospitals, is not being uniformly implemented. The guideline indicated that the major avoidable factors at the primary healthcare clinics were inaccurate assessments and misdiagnosis, although tertiary hospitals also failed to adhere to protocols.^9^ Furthermore, we note that rural areas and public health sectors, which cater for the majority of South Africans, face challenges with access to healthcare services, equipment, limited re-usable resources in available clinics, shortage of specialised healthcare professionals, such as family physicians, and referral delays because of limited emergency medical services.^2^

## Prevention strategies

At the primary care level, equipment needs to be available and functional, for example, validated blood pressure devices and urine-dipstick supplies. In addition, there should be regular training for all healthcare professionals,^10^ especially to identify women at risk of HDP (such as obese women and those over 35 years of age).^9^ Most importantly, regular training of midwives in the management of HDPs is required.^11^ At a system level, primary care facilities require a reliable supply chain for essential medicines, clear referral pathways, and rapid referral transport mechanisms. Furthermore, regular clinical audits with feedback on frontline teams are crucial.^12^

## Conclusion

Healthcare services for HDP need to improve in primary care clinics to reduce avoidable causes of maternal deaths. This editorial recommends that family physicians, general practitioners, frontline nursing professionals and midwives must use recommended simple, consistent practices for HDP screening, initial management, and referral pathways to reduce avoidable maternal mortalities and stillbirths.
